# Predictive Value of MiR-219-1, MiR-938, MiR-34b/c, and MiR-218 Polymorphisms for Gastric Cancer Susceptibility and Prognosis

**DOI:** 10.1155/2017/4731891

**Published:** 2017-02-19

**Authors:** Yanhua Wu, Zhifang Jia, Donghui Cao, Chuan Wang, Xing Wu, Lili You, Simin Wen, Yuchen Pan, Xueyuan Cao, Jing Jiang

**Affiliations:** ^1^Division of Clinical Research, First Hospital of Jilin University, Changchun, Jilin Province 130021, China; ^2^Maternal and Child Health Center of Chaoyang District, Beijing 100020, China; ^3^Department of Gastrointestinal Surgery, First Hospital of Jilin University, Changchun 130021, China

## Abstract

Gastric cancer (GC) is one of the most prominent global cancer-related health threats. Genes play a key role in the precise mechanisms of gastric cancer. SNPs in mi-RNAs could affect mRNA expression and then affect the risk and prognosis of GC. Firstly, we have decided to perform a case-control study which included 897 GC patients and 992 controls to evaluate the association of miR-219-1 rs213210, miR-938 rs2505901, miR-34b/c rs4938723, and miR-218 rs11134527 polymorphisms with gastric cancer susceptibility. Secondly, among the 897 GC patients above, 755 cases underwent a radical operation, without distant metastasis and with negative surgical margins included in the survival analysis to evaluate the association of the four SNPs above with gastric cancer prognosis. The C/T or C/C genotypes of rs213210 were related to a lower GC risk (OR = 0.76, 95% CI: 0.62–0.93, *P* = 0.009) compared to the T/T genotype. Rs11134527 in miR-218 was associated with GC survival, and the G/A and G/G genotypes of rs11134527 resulted in a decreased risk of death when compared with the A/A genotype (HR = 0.75, 95% CI: 0.61–0.95, *P* = 0.016). This study found that miR-219-1 rs213210 polymorphism was associated with GC susceptibility and rs11134527 in miR-218 was positively correlated with GC prognosis.

## 1. Introduction

Gastric cancer (GC) is one of the most prominent global cancer-related health threats [1, 2]. A recent report from the National Central Cancer Registry of China showed that GC was the second most common malignant cancer and the third most common cause of cancer-related death in China [3].

MicroRNAs (miRNA) area class of evolutionarily conserved small noncoding RNA that negatively regulates gene expression at a posttranscriptional level by inhibiting translation through binding to the complementary site of a targeted mRNA [4, 5]. Aberrant expression of miRNA may characterize many diseases, including cancer, and these patterns could be used as predictors of susceptibility and prognosis in cancer patients [6, 7]. Various studies have indicated that single nucleotide polymorphisms (SNPs) in the 3′-untranslated region affected the expression levels or the mature sequence of miRNAs and further impacted mRNA and protein expressions [8–10].

Various studies linked miRNA genetic variations in both miRNA encoding genes and in their target genes to GC risk and prognosis. Single nucleotide polymorphisms were the common genetic variations; SNPs in miRNAs could affect mRNA expression by altering the transcription of the primary miRNA transcript and the interaction of miRNA with mRNA [11, 12]. SNPs in different candidate miRNAs (i.e., let-7e rs8111742, miR-365b rs121224, miR-4795 rs1002765, miR-499 rs3746444, and miR-146a rs2910164) have been associated with the susceptibility or prognosis of GC [10, 13, 14]. More recently, Arisawa et al. found that rs2505901 in miRNA-938 was associated with susceptibility to gastric cancer in a Japanese population [15]. Other studies found that miR-34b/c rs4938723 polymorphism was associated with breast cancer and cervical cancer risk [16, 17]. A study by Pardini et al. showed that rs213210 in miR-219-1 was associated with clinical outcomes of colorectal cancer patients [18] and a meta-analysis indicated that miR-218 rs11134527 polymorphism may have an association with many types of cancer [19].

Some of the SNPs above have been studied in some other cancers or populations, but had not been examined in Chinese Han population with regard to gastric cancer. In the present study, we took advantage of these interesting results to investigate whether miR-219-1 rs213210, miR-938 rs2505901, miR-34b/c rs4938723, and miR-218 rs11134527 polymorphisms were related to the risk and clinical outcomes of gastric cancers in a Chinese Han population.

## 2. Materials and Methods

### 2.1. Study Population

Gastric cancer patients were enrolled from July 2008 to December 2013 in the Gastric and Colorectal Surgery Department of the First Hospital, Jilin University, Changchun, China. All cases were histologically diagnosed as GC by a pathologist, and no patients had received preoperative chemotherapy or radiotherapy. In total, 897 GC patients were included in the association study between SNPs and GC development. Additionally, tumor-free controls were recruited from the Physical Examination Center in the First Hospital of Jilin University. In all, 992 frequency age- (±5 years) and gender-matched control participants were included in the study.

**Figure 1:**
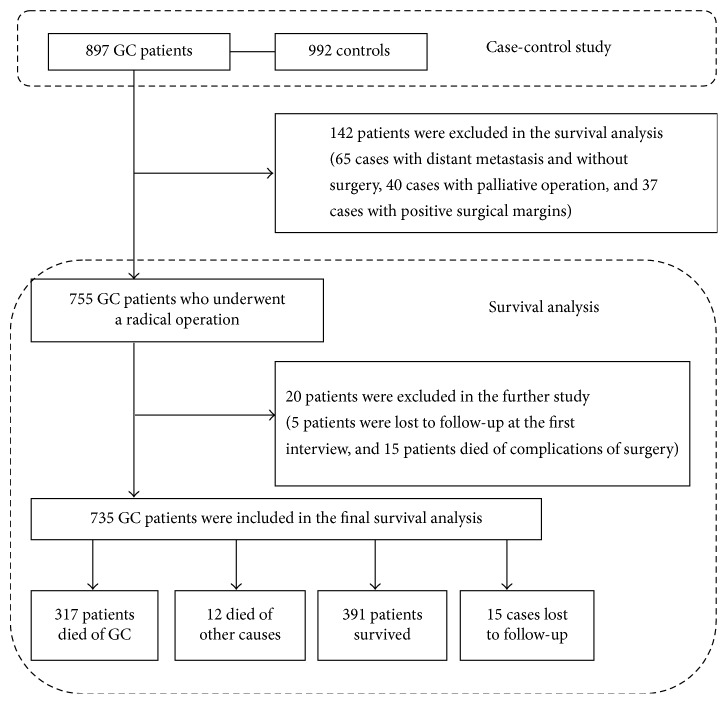
The flow chart of study design.

Patients with distant metastasis and positive surgical margins were more susceptible to worse outcomes. To assess the association between SNPs and longer survival status in GC patients who underwent tumor curative resection, patients with distant metastasis and without surgery, with palliative operation, and with positive surgical margins were excluded in the survival analysis. As a result, 755 patients were included in the survival analysis within the 897 GC patients mentioned above, and the flow chart of our study design was shown in Figure 1. The study was approved by the ethics committee of the First Hospital of Jilin University. All subjects in this study signed a written informed consent form.

### 2.2. Data Collection

Clinicopathologic parameters (including tumor sizes, histological grade, WHO classification, vascular invasion, neural invasion, depth invasion, lymph metastasis, clinical stages, and postoperational chemotherapy) were collected from the medical records. The pathological classification of tumors was determined according to the AJCC/UICC, 2010 classifications. Postoperational chemotherapy was defined as at least three cycles of chemotherapy that were received after surgery and that were divided into FOLFOX-4 (a combination of 5-fluorouracil, leucovorin and oxaliplatin); XELOX (a combination of capecitabine and oxaliplatin); other (such as capecitabine or 5-fluorouracilalone); or none.

Gastric cancer patients were followed up on the third month, sixth month, and first year after the tumorectomy and every subsequent year until death or the end of our study. Survival time was defined as the duration from the date of surgery to the date of death (if patients died) or to the date of the last successful interview (if patients were alive or lost to follow-up). Patients with palliative operation, distant metastasis, or positive surgical margins were excluded in the survival analysis. Patients who died from complications of the surgical operation in the preoperative period or were lost to follow-up at the first time of interview were also excluded from the survival analysis.

### 2.3. Tests of* Helicobacter pylori* (*H. pylori*) Infection

Enzyme-linked immune absorbent assay kit (Biohit, Finland) was used to determinate the* H. pylori *infection. Titers higher than 30 EIU were classified as positive for* H. pylori* infection according to the manufacturer's instruction.

### 2.4. Genotyping

Four SNPs in miRNAs (miR-219-1 rs213210, miR-938 rs2505901, miR-34b/c rs4938723, and miR-218 rs11134527) were selected based on previous studies that documented associations between SNPs and GC or other cancers in other ethnicities. The minor allele frequency (MAF) of all the SNPs was greater than 0.05 in the Chinese Han population.

Blood samples of GC cases were collected before the gastric cancer resection, and fasting blood samples of controls were collected during the same period with GC groups. Both case and control blood samples were stored at −80°C in EDTA tubes. Genomic DNA was extracted following the manufacturer's instructions (AxyPrep Blood Genomic DNA Miniprepkit, Axygen, Union City, CA, USA). Genotyping of each SNP was conducted using the MassARRAY technology platform (Sequenom, CA, USA) and determined by BGI tech (Beijing, China). The detection rates for the SNPs miR-219-1 rs213210, miR-938 rs2505901, miR-34b/c rs4938723, and miR-218 rs11134527 were 99.3%, 99.7%, 99.6%, and 99.7%, respectively. 1% of samples were randomly selected and tested repeatedly, and the concordance rates were 100% for all the four SNPs.

### 2.5. Statistical Analyses

Continuous variables with a normal distribution were described as the mean ± standard deviation and compared with Student's *t*-test. Discrete variables were described as frequency (percentage) and compared using the *χ*^2^ test. For each SNP, test of the Hardy-Weinberg disequilibrium (HWD) was conducted. Associations between the SNPs and GC were computed using logistic regression model adjusted for age, gender, and* H. pylori* infection. The Kaplan–Meier method was used to plot the survival curves, and the log-rank test was conducted for comparing the survival curves between different genotypes within each SNP. Univariate and multivariate Cox regression models were performed to assess the hazard ratios (HRs) and 95% CIs of the possible prognostic factors. All of the analyses were conducted using the SPSS program (version 17.0, Chicago, IL, USA), and *P* < 0.05 was considered to be statistically significant.

## 3. Results

### 3.1. Subject Characteristics

A total of 1889 participants (897 gastric cancer cases and 992 tumor-free controls) were enrolled in our study. The characteristics of the subjects were shown in Table 1. The gender ratio between the gastric cancer group and the control group was not significantly different (*P* = 0.898). As a result of frequency matching by age (±5 years), the average age was two years younger in the control group compared to the GC group. The* H. pylori*-positive rate was significantly higher in GC patients than that in controls (*P* < 0.05).

### 3.2. Allele Frequency Comparisons

The distributions of 4 SNPs (rs213210, rs2505901, rs4938723, and rs11134527) were all in Hardy-Weinberg equilibrium (HWE) in the control group. The comparisons of genotype distributions and allele frequencies of the 4 SNPs between GC and controls were shown in Table 1. Allele frequency differences between the two groups were not observed for any of the 4 SNPs. A significant difference was only revealed in the rs213210 genotype distributions between the two groups (*P* = 0.020). Additionally, in the control group, the C/C genotype of rs213210 was associated with lower risk of* H. pylori* infection (OR = 0.66, 95% CI: 0.46–0.95, *P* = 0.025, Table S1 in Supplementary Material available online at https://doi.org/10.1155/2017/4731891).

### 3.3. Association of SNPs with Risk of Gastric Cancer

Genotype distribution of codominant and dominant models of four SNPs was shown in Table 2. Compared to the T/T genotype, the C/T or C/C genotypes of rs213210 were associated with a lower GC risk (OR = 0.76, 95% CI = 0.62–0.93, *P* = 0.009) after adjusting for the confounding factors of age, gender, and* H. pylori* infection.

### 3.4. Association between SNPs and Survival of Gastric Cancer

Among the 897 GC patients mentioned above, 65 cases were with distant metastasis and without surgery, 40 cases with palliative operation, and 37 cases with positive surgical margins. These cases were excluded from the survival analysis. Among the remaining 755 cases, 5 patients were lost to follow-up at the first interview, 15 patients died of complications of surgery, and these 20 (2.2%) cases were also excluded from survival analysis. Finally, 735 patients were included in the final survival analysis. The median follow-up time was 51.64 months (ranging from 1.61 to 94.32 months). During the follow-up, 317 (43.1%) patients died of GC, 12 (1.6%) died of other causes, 391 (53.2%) patients survived, and 15 (2.0%) cases were lost to follow-up; see Figure 1.

**Figure 2:**
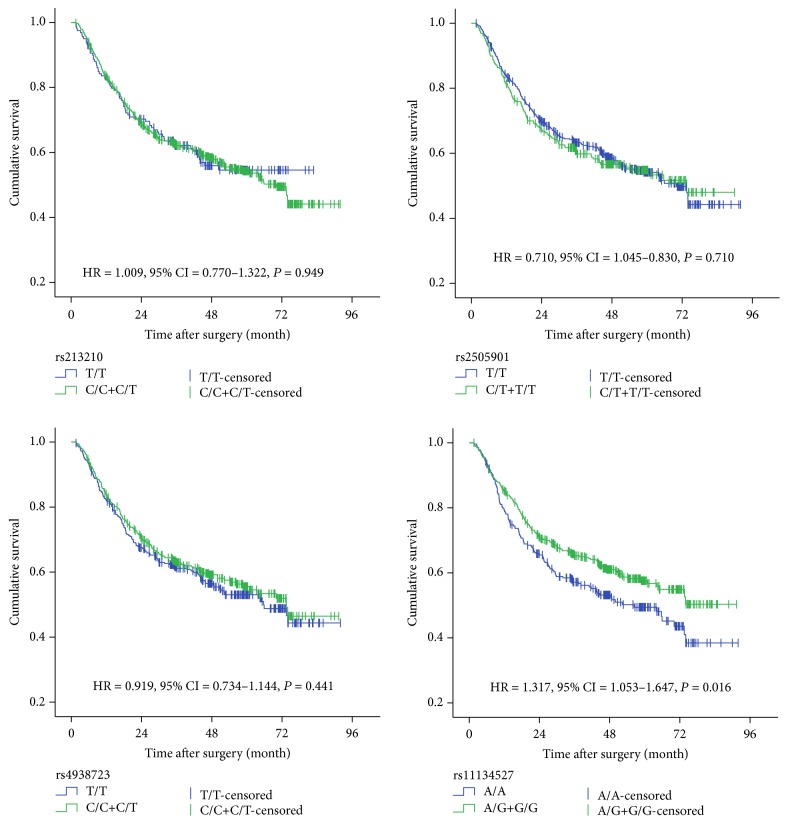
Survival plots for SNPs in miRNAs using the dominant model of gastric cancer patients.

Survival analysis was performed to evaluate the associations of clinicopathologic parameters and SNP genotypes on survival of gastric cancer using the dominant model. As shown in Table 3, tumor size ≥5 cm, high histological grade, T3/T4 depth of invasion, N1/N2/N3 lymph metastasis, and higher TNM stage were associated with worse survival of GC. In this study, 48.5% of patients carrying the rs11134527 A/A genotype died of GC compared to only 39.5% of cases with A/G-G/G genotypes. Patients bearing genotypes A/G-G/G of rs11134527 were found to live longer than those bearing genotype A/A (HR = 0.75, 95% CI: 0.61–0.95, *P* = 0.016) (Figure 2). No significant associations were observed between SNPs and other clinic pathological parameters such as tumor size, histological grade, WHO classification, TNM stage, distant metastasis, and chemotherapy (data not shown).

As shown in Table 4, the results from the multivariate Cox regression test showed that rs11134527 was an independent prognostic factor. Patients carrying G/A or G/G genotypes lived longer than patients with the A/A genotype (HR = 0.73, 95% CI: 0.58–0.93, *P* = 0.010). Additionally, positive vascular invasion, tumor size ≥5 cm, and higher TNM stage (II and III) were also independently associated with worse prognosis. Receiving standard XELOX chemotherapy after tumor resection was associated with better OS (HR: 0.51, 95% CI: 0.33–0.80, *P* = 0.003).

## 4. Discussion

In the present work, we assessed the association between four SNPs in miRNAs and susceptibility or prognosis in gastric cancer patients. Among them, rs213210 in miR-219-1 was associated with the risk of GC. Compared to the T/T genotype, patients carrying the C/T and C/C genotypes of rs213210 had a lower GC risk. On the other hand, rs11134527 in miR-218 was associated with GC survival; the G/A or G/G genotypes of rs11134527 resulted in a decreased risk of death when compared with the A/A genotype.

MicroRNAs are involved in various crucial biological processes by targeting hundreds of mRNAs that take part in cell proliferation, differentiation, apoptosis, and the progression of cancer [20]. Single nucleotide polymorphisms in miRNAs could alter the transcription of the primary miRNA transcript and the interaction of miRNA with mRNA, thus affecting mRNA expression [11, 12]. Moreover, researchers have found that half of the miRNA genes may be located in cancer-related regions [21]. As a result, SNPs in these miRNAs could be associated with cancer risk or prognosis [22]. Though there were many GWAS results have been published, but they always focused on the SNPs on coding genes, not on miRNA, and refer only to the cancer risk but not the cancer prognosis. The present study aimed to assess the association between miR-219-1 rs213210, miR-938 rs2505901, miR-34b/c rs4938723, and miR-218 rs11134527 polymorphisms not only with GC susceptibility but also with GC prognosis.

For rs213210 in miR-219-1, only a few studies have investigated the role of this polymorphism in cancer susceptibility. Song et al. reported that there was no association between the rs213210 polymorphism and risk of esophageal squamous cell carcinoma in Chinese [23]. Yoon, K. A et al. found that no significant association emerged between this polymorphism and survival of nonsmall cell lung cancer. Further, Pardini et al. thought that rs231210 could be a predictor of clinical outcomes in colorectal cancer patients [8]. However, our results suggested that rs231210 was associated with GC susceptibility but not associated with GC prognosis. To some extent, it was not suitable to compare the results of the prior studies to ours since there were different ethnicities and cancer categories involved in each. We used F-SNP online software (http://compbio.cs.queensu.ca/F-SNP/) to predict the function of rs231210 and found that this SNP is located in a region involved in transcriptional regulation. The change from T to C of rs231210 could have an effect upon the expression of miR-219-1. Research on the targeting gene of miR-219-1 found that mature miR-219-1 is involved in the pathway related to transcription regulation, regulation of RNA metabolic processes, transcription from RNA polymerase II promoter, and gene expression [18]. Another study indicated that upexpression of miR-219-1-3p induced a decrease of cell proliferation and migration in pancreatic cancer by negatively regulating expression of the mucin MUC4. The studies could partially confirm the importance of the SNPs in miR-219-1-related genes in the mechanisms of cancer risk.

The association between rs11134527 in miR-218 and cancer risk is tumor location-dependent. A meta-analysis showed that rs11134527 polymorphism was associated with risk of cervical cancer but not hepatocellular carcinoma [24] In the present study, no association was observed between rs11134527 and GC risk in all of the four genetic models (dominant model, recessive model, codominant model, and overdominant model). Jiang et al. found that the G/G genotype of rs11134527 was significantly associated with a better prognosis of esophageal squamous cell carcinoma in a codominant model [25]. In our study, we used the dominant model to assess the association between rs11134527 and GC and found that people carrying the G allele lived longer. Then, we used online software to search the target gene of miR-218. Finally, twenty genes were validated according to the miRWalk tool [26]. Some of the target genes were closely related to cancer development, such as CDH2, ZEB2, MAP3K2, and SLC24A4. Other target genes of miR-218 that have been identified by previous researchers including BMI1, LAMB3, and LASP1 and the expressions of these genes were associated with the invasion or prognosis in different cancer types [27–29]. In addition to this, we evaluated the expression levels of miR-218 among 359 GC patients in the TCGA dataset and observed that higher miR-218 expression level was associated with a better survival of GC (*P* = 0.002). Recent research also confirmed that lower miR-218 tissue expression level was associated with aggressive progression of gastric cancer [30]. Meanwhile, miR-218 could inhibit invasion and metastasis of gastric cancer by targeting the Robo1 receptor [31]. RNAfold prediction analysis showed that the transition from A to G of rs11134527 could alter the local second structure of miR-218 [32]. Thus, we supposed that the expression level of miR-218 may be partly relative to SNPs and more researches are needed in the future.

Another study found that rs2505901 in miR-938 was associated with susceptibility to gastric cancer in a Japanese population [15] and other studies found that miR-34b/c rs4938723 polymorphism was associated with risk of breast cancer and cervical cancer. However, the present study indicated that miR-938 rs2505901 and miR-34b/c rs4938723 were associated neither with gastric cancer nor with susceptibility or with prognosis. The different cancer types, ethnicities, and sample sizes might contribute to these discrepancies.

## 5. Conclusions

In conclusion, rs213210 in miR-219-1 was associated with the risk of GC. Compared to the T/T genotype, the C/T and C/C genotypes of rs213210 were associated with a lower GC risk. On the other hand, rs11134527 in miR-218 was associated with GC survival in patients without distant metastasis and without positive surgical margins. The G/A and G/G genotypes of rs11134527 resulted in decreased risk of death when compared with the A/A genotype. More laboratory study is needed to clarify the underlying mechanism of SNPs in miRNAs.

## Supplementary Material

Relationship of *H. pylori* infection and SNPs in the control group shown that, the C/C genotype of re213210 was associated with lower risk of *H. pylori* infection (OR = 0.66, 95% CI: 0.46-0.95, *P* = 0.025).

## Figures and Tables

**Table 1:** Characteristics, genotype distributions, and allele frequencies of SNPs between GC and controls.

Characteristics	Gastric cancer (*n* = 897)	Controls (*n* = 992)	*P*
Age	61.04 ± 11.34	59.15 ± 10.04	**<0.001**
Gender			
Male	648 (72.2%)	714 (72.0%)	0.898
Female	249 (27.8%)	278 (28.0%)	
*H. pylori* infection			
Yes	603 (67.6%)	487 (49.1%)	**<0.001**
No	289 (32.4%)	505 (50.9%)	
Rs213210			
T/T	266 (30.0%)	241 (24.4%)	**0.020**
C/T	422 (47.6%)	519 (52.5%)	
C/C	199 (22.4%)	229 (23.1%)	
T	954 (53.8%)	1001 (50.6%)	0.052
C	820 (46.2%)	977 (47.9%)	
Rs2505901			
T/T	587 (65.5%)	635 (64.3%)	0.853
C/T	274 (30.6%)	313 (31.7%)	
C/C	35 (3.9%)	40 (4.0%)	
T	1448 (80.8%)	1583 (80.1%)	0.593
C	344 (19.9%)	393 (19.9%)	
Rs4938723			
T/T	405 (45.4%)	476 (48.1%)	0.287
C/T	396 (44.3%)	430 (43.4%)	
C/C	92 (10.3%)	84 (8.5%)	
T	1206 (67.5%)	1382 (69.8%)	0.133
C	580 (32.5%)	598 (30.2%)	
Rs11134527			
A/A	345 (38.6%)	394 (39.8%)	0.737
A/G	412 (46.1%)	439 (44.3%)	
G/G	137 (15.3%)	158 (15.9%)	
A	1102 (61.6%)	1227 (61.9%)	0.867
G	686 (38.4%)	755 (38.2%)	

Data were described as mean ± SD or *N* (%).

**Table 2:** Genotype distribution comparisons and odds ratio (OR) estimates of 4 SNPs for GC.

Genotype	Case	Control	OR (95% CI)^a^	*P* ^a^
Rs213210				
T/T	266 (30.0%)	241 (24.4%)	1.00	**0.026**
C/T	422 (47.6%)	519 (52.5%)	0.74 (0.60–0.92)	
C/C	199 (22.4%)	229 (23.1%)	0.80 (0.62–1.04)	
C/T-C/C	621 (70.0%)	748 (75.6%)	0.76 (0.62–0.93)	**0.009**
Rs2505901				
T/T	587 (65.5%)	635 (64.3%)	1.00	0.870
C/T	274 (30.6%)	313 (31.7%)	0.95 (0.78–1.16)	
C/C	35 (3.9%)	40 (4.0%)	0.93 (0.58–1.49)	
C/T-C/C	309 (34.5%)	353 (35.7%)	0.95 (0.79–1.15)	0.600
Rs4938723				
T/T	405 (45.4%)	476 (48.1%)	1.00	0.230
C/T	396 (44.3%)	430 (43.4%)	1.09 (0.90–1.32)	
C/C	92 (10.3%)	84 (8.5%)	1.32 (0.95–1.82)	
C/T-C/C	488 (54.6%)	514 (51.9%)	1.13 (0.94–1.35)	0.200
Rs11134527				
A/A	345 (38.6%)	394 (39.8%)	1.00	0.700
A/G	412 (46.1%)	439 (44.3%)	1.07 (0.88–1.31)	
G/G	137 (15.3%)	158 (15.9%)	0.97 (0.74–1.28)	
A/G-G/G	549 (61.4%)	597 (60.2%)	1.05 (0.87–1.26)	0.063

ORs and *P* values were adjusted by age, gender, and *H. pylori *infection using logistic regression model. Here, a means adjusted *P* value.

**Table 3:** Univariate analysis for prognostic factors of gastric cancer.

Characteristic	Patient *n*	Death *n* (%)	Mean OS^*∗*^ (months)	*P* ^†^
Age (year)				
≤45 years	65	29 (44.6)	54.01	0.935
>45 years	670	287 (42.8)	57.42	
Gender				
Male	539	237 (44.0)	56.55	0.343
Female	196	79 (40.3)	59.35	
Tumor size				
<5 cm	422	144 (34.1)	64.89	<0.001
≥5 cm	302	165 (54.6)	47.22	
Histological grade				
low grade	309	118 (38.2)	61.85	0.006
high grade	373	177 (47.5)	52.74	
WHO classification				
Tubular	569	238 (41.8)	58.55	0.167
Signet-ring cell	36	18 (50.0)	50.57	
Others	130	60 (46.2)	45.23	
Vascular invasion				
Negative	217	39 (18.0)	76.06	<0.001
Positive	506	270 (53.4)	49.61	
Neural invasion				
Negative	330	97 (29.4)	68.19	<0.001
Positive	393	212 (53.9)	48.43	
Depth of invasion				
T1/T2	198	26 (13.1)	80.23	<0.001
T3/T4	515	278 (54.0)	49.00	
Lymph metastasis				
N0	214	33 (15.4)	76.15	<0.001
N1/N2/N3	501	271 (54.1)	48.79	
TNM stage				
I	136	12 (8.8)	81.13	<0.001
II	278	86 (30.9)	67.90	
III	312	212 (67.9)	36.55	
Chemotherapy				
None	486	200 (41.2)	57.68	0.056
FOLFOX-4	137	66 (48.2)	52.37	
XELOX	74	26 (35.1)	62.25	
Others	38	24 (63.2)	42.63	
Rs213210				
T/T	159	67 (42.1)	54.24	0.949
C/C+C/T	566	245 (43.3)	57.14	
Rs2505901				
T/T	476	204 (42.9)	57.52	0.710
C/T-C/C	258	112 (43.4)	56.11	
Rs4938723				
T/T	330	148 (44.8)	65.71	0.440
C/C-C/T	401	166 (41.4)	73.50	
Rs11134527				
A/A	276	134 (48.5)	52.93	0.016
A/G-G/G	456	180 (39.5)	60.01	

^*∗*^For most characteristics, less than half patients were dead, so mean overall survival (OS) time was presented for most of the median OS that could not be calculated. ^†^*P* values were computed by the log-rank test.

**Table 4:** Multivariate Cox regression test for prognostic factors of gastric cancer.

Variable	HR	95% CI	*P*
Vascular invasion			
Negative	1	—	—
Positive	1.90	1.27–2.76	0.002
Tumor size			
<5 cm	1	—	—
≥5 cm	1.41	1.11–1.80	0.005
TNM stage			
I	1	—	—
II	2.86	1.44–5.67	0.003
III	9.60	4.82–19.02	<0.001
Chemotherapy			
None	1	—	—
FOLFOX-4	0.90	0.67–1.20	0.444
XELOX	0.51	0.33–0.80	0.003
Others	0.99	0.64–1.56	0.988
Rs11134527			
A/A	1	—	—
G/A-G/G	0.73	0.58–0.93	0.010

HR: Hazard ratio. *P* values were calculated with multivariate Cox regression with the stepwise selection method including all the variables at initiation.
