# Supplementary material for: Predictive Value of MiR-219-1, MiR-938, MiR-34b/c, and MiR-218 Polymorphisms for Gastric Cancer Susceptibility and Prognosis
Source: Dis Markers. 2017 Feb 19;2017:4731891. doi: 10.1155/2017/4731891 (PMC5337385; doi:10.1155/2017/4731891)
Supplement: Supplementary file 1 — Relationship of H. pylori infection and SNPs in the control group shown that, the C/C genotype of re213210 was associated with lower risk of H. pylori infection (OR = 0.66, 95% CI: 0.46-0.95, P = 0.025). [file 4731891.f1.pdf]

Table S1. Relationship of *H.pylori* infection and SNPs in the control group

Genotype	*H.pylori*$^+$ (N=485)	*H.pylori*$^-$ (N=504)	OR(95%CI)[a]	*P* value
Rs213210				
T/T	123(25.4%)	106(21.0%)	1	
C/T	258(53.2%)	261(51.8%)	0.835(0.625-1.165)	0.318
C/C	104(21.4%)	137(27.2%)	**0.660(0.458-0.950)**	**0.025**
Rs2505901				
T/T	20(4.1%)	20(4.0%)	1	
C/T	159(32.7%)	154(30.7%)	1.016(0.525-1.964)	0.963
C/C	307(63.2%)	328(65.3%)	0.920(0.485-1.746)	0.800
Rs4938723				
T/T	49(10.1%)	35(6.9%)	1	
C/T	214(44.0%)	216(42.9%)	0.714(0.445-1.147)	0.163
C/C	223(45.9%)	253(50.2%)	0.629(0.393-1.007)	0.054
Rs11134527				
A/A	198(40.7%)	196(38.8%)	1	
A/G	211(43.4%)	228(45.1%)	0.917(0.698-1.204)	0.534
G/G	77(15.8%)	81(16.0%)	0.948(0.655-1.372)	0.778

[a]ORs were calculated adjusting for age and sex in logistic regression model.

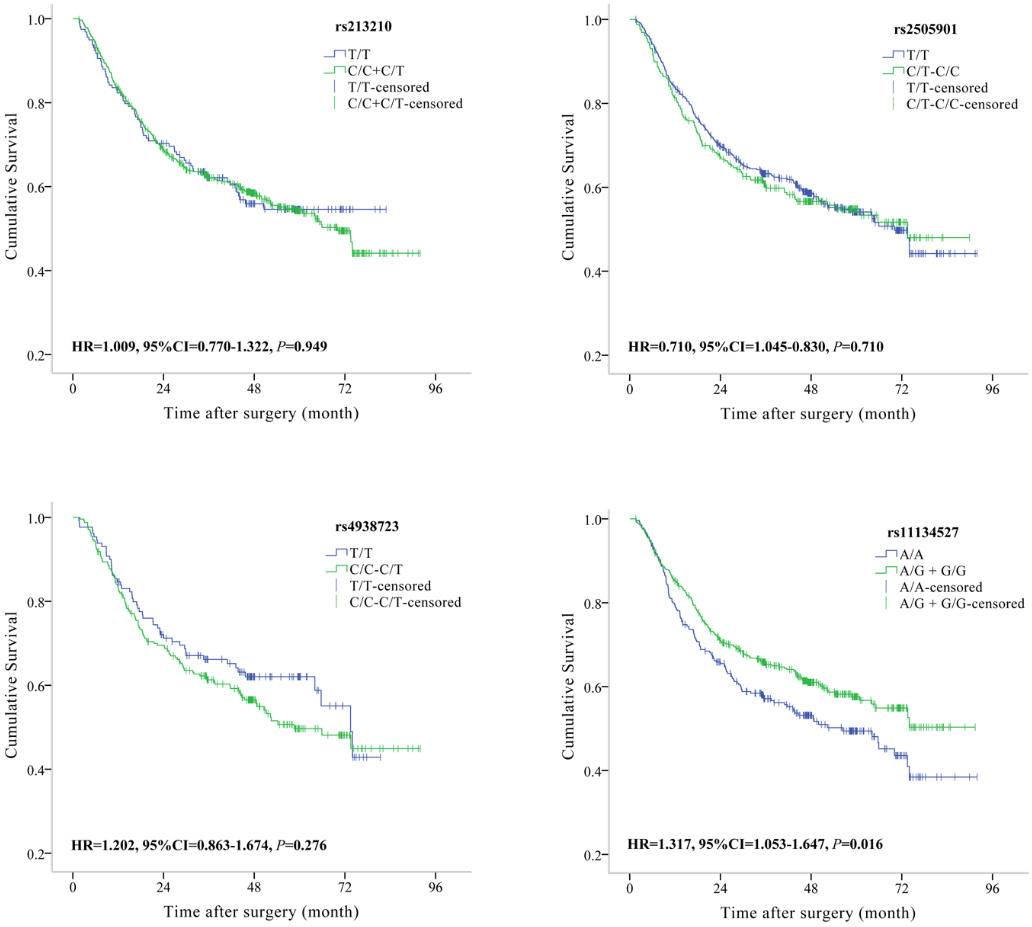
rs213210
T/T
C/C+C/T
T/T-censored
C/C+C/T-censored
HR=1.009, 95%CI=0.770-1.322, P=0.949
Cumulative Survival
Time after surgery (month)
rs2505901
T/T
C/T-C/C
T/T-censored
C/T-C/C-censored
HR=0.710, 95%CI=1.045-0.830, P=0.710
Cumulative Survival
Time after surgery (month)
rs4938723
T/T
C/C-C/T
T/T-censored
C/C-C/T-censored
HR=1.202, 95%CI=0.863-1.674, P=0.276
Cumulative Survival
Time after surgery (month)
rs11134527
A/A
A/G + G/G
A/A-censored
A/G + G/G-censored
HR=1.317, 95%CI=1.053-1.647, P=0.016
Cumulative Survival
Time after surgery (month)
